# Characterizing hemodynamic response alterations during basketball dribbling

**DOI:** 10.1371/journal.pone.0238318

**Published:** 2020-09-03

**Authors:** Daniel Carius, Oliver Seidel-Marzi, Elisabeth Kaminski, Niklas Lisson, Patrick Ragert

**Affiliations:** 1 Institute for General Kinesiology and Exercise Science, University of Leipzig, Leipzig, Germany; 2 Max Planck Institute for Human Cognitive and Brain Sciences, Leipzig, Germany; Washington University in Saint Louis School of Medicine, UNITED STATES

## Abstract

Knowledge on neural processing during complex non-stationary motion sequences of sport-specific movements still remains elusive. Hence, we aimed at investigating hemodynamic response alterations during a basketball slalom dribbling task (BSDT) using multi-distance functional near-infrared spectroscopy (fNIRS) in 23 participants (12 females). Additionally, we quantified how the brain adapts its processing as a function of altered hand use (dominant right hand (DH) vs. non-dominant left hand (NDH) vs. alternating hands (AH)) and pace of execution (slow vs. fast) in BSDT. We found that BSDT activated bilateral premotor cortex (PMC), supplementary motor cortex (SMA), primary motor cortex (M1) as well as inferior parietal cortex and somatosensory association cortex. Slow dominant hand dribbling (DH_slow_) evoked lower contralateral hemodynamic responses in sensorimotor regions compared to fast dribbling (DH_fast_). Furthermore, during DH_slow_ dribbling, we found lower hemodynamic responses in ipsilateral M1 as compared to dribbling with alternating hands (AH_slow_). Hence, altered task complexity during BSDT induced differential hemodynamic response patterns. Furthermore, a correlation analysis revealed that lower levels of perceived task complexity are associated with lower hemodynamic responses in ipsilateral PMC-SMA, which is an indicator for neuronal efficiency in participants with better basketball dribbling skills. The present study extends previous findings by showing that varying levels of task complexity are reflected by specific hemodynamic response alterations even during sports-relevant motor behavior. Taken together, we suggest that quantifying brain activation during complex movements is a prerequisite for assessing brain-behavior relations and optimizing motor performance.

## Introduction

While brain processing and its functional plasticity during the execution of stationary, simplified movements is well characterized [1–4], little is known about brain regions during the execution of complex sport-specific skills. To date, the aforementioned studies have investigated task-related changes in brain functioning during the execution of motor tasks, mostly using simple and/or complex finger sequences. These studies suggest, that task complexity is an important feature which systematically changes neural activation during movement execution. Task complexity can be varied on many different levels, for example by increasing or reducing pace of execution [5–8] but also by experimentally inducing laterality effects [3, 4, 9–11].

In detail, comparative analyses of movement pace revealed heterogeneous findings for finger movements. For example, when comparing slow vs. fast finger tapping (up to 4 Hz), no differential levels of cortical activation were found [5, 12]. Kuboyama et al. [6] added, that the sensorimotor cortex (SMC) only showed differential activation during tapping with maximum effort (> 4 Hz). By contrast, investigation of laterality effects on simple finger [3, 4, 9] and hand movements [1, 3, 10] provided more consistent findings. For example, participants performing a power grip task with both, their dominant and non-dominant hand, showed activation in sensorimotor areas in both conditions with additional activation of subcortical and cerebellar brain regions for the non-dominant hand condition, indicating distinct contributions of brain networks as a function of laterality [10].

Apart from this evidence on simple motor tasks, knowledge on brain functioning during the execution of complex sport-specific movements and task-related functional adaptations is still limited [13–17]. It is by no means clear, whether previous knowledge from simple movements is transferable to complex/ whole-body movements. This, however, is particularly of interest, since a better characterization of brain functioning during sport-specific movements could be beneficial on several levels [18]. On the one hand, a profound understanding of brain functioning during complex movements and associated behavioral parameters might help to systematically guide training processes and to optimize training outcomes. On the other hand, identifying performance- and task-related brain areas is a prerequisite for targeted neuromodulation to augment motor performance and/or sport-specific skills.

Hence, the present study aimed at investigating task-related changes in hemodynamic response alterations during a complex sport-specific movement using multi-distance fNIRS. Sport-specific movement was assessed using a complex basketball slalom dribbling task (BSDT), where task complexity was systematically varied using different levels of hand use and movement pace. Based on previous studies, we hypothesized that (a) brain regions involved in motor planning, preparation and execution such as primary motor cortex (M1), premotor cortex (PMC) and supplementary motor cortex (SMA) are involved in BSDT [2, 10, 19]. Furthermore, we expected (b) lower hemodynamic responses during slow dribbling as compared to fast dribbling conditions [5, 7, 20]. Considering laterality effects, we hypothesized that (c) dribbling with the dominant right hand (DH) induces lower ipsilateral hemodynamic responses compared to dribbling with the non-dominant left hand (NDH) [3, 10, 11], and that (d) dribbling with DH induces lower hemodynamic responses compared to dribbling with alternating hands (AH). Additionally, we hypothesized that (e) the amount of change in hemodynamic responses would relate to the perceived level of task complexity.

## Material and methods

### Subjects

A total number of 23 healthy volunteers (mean age: 24.61 ± 0.47 years; range 21–29 years; 12 females) were enrolled in the present study (see Table 1 for characteristics). Study procedures were approved by the local ethics committee of the University of Leipzig. The study was performed in accordance with the Declaration of Helsinki. None of the participants had a history of neurological illness, and during the time of the experiment, none of the volunteers was taking any central-acting drugs. All volunteers were right-handed according to the Edinburgh Handedness Questionnaire (mean handedness score of 83.34 ± 3.05; [21]). Total hours of sports per week as well as duration of fine-motor training per week (e.g., playing a musical instrument, knitting, doing handcrafts, playing video games) were assessed with a questionnaire. None of the participants stated to have ever played basketball on a regular basis. Before and after the entire experimental procedure, all participants rated their levels of attention, fatigue and discomfort on a visual analog scale (VAS) to rule out unspecific effects of these parameters biased the study results.

**Table 1:** Group demographics.

Group	Age (years)	Gender (female/ male)	LQ	Sports/week (hours)	Fine-motor training/ week (hours)
*n* = 23	24.61 ± 0.47	12/11	83.34 ± 3.05	5.09 ± 0.64	1.65 ± 0.60

LQ, Laterality Quotient as assessed with the Edinburgh Handedness Scale [range: -100 (full left-handed) to +100 (full right-handed)]. Hours of sports per week and hours of fine motor training per week (e.g., playing a musical instrument, knitting, doing hand crafts, playing videogames with keypad or joystick) were assessed with a questionnaire. All values are depicted as mean standard error (SE) of the mean.

### Experimental procedure

**Fig 1:**
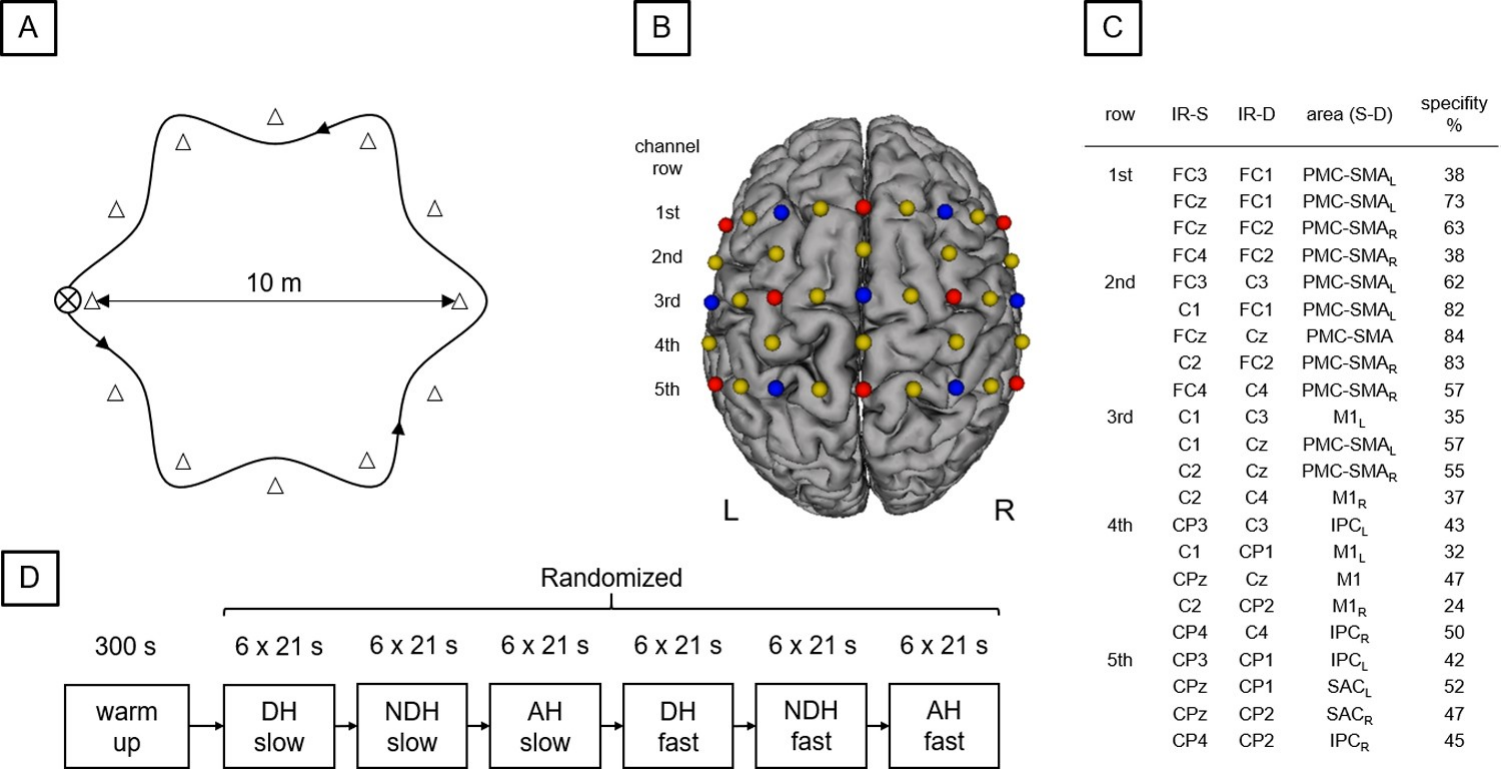
Study design and experimental setup. A: Slalom course with a diameter of 10 m including 12 slalom bars. The distance between the bars is approx. 2.60 m each. B: Illustration of fNIRS configuration used during BSDT. Sources are shown as red dots and detectors as blue dots. Yellow dots represent each center of the 22 channels (inter-optode distance 3 cm). C: 10–20 positions for infrared sources (IR-S) and detectors (IR-D), respective brain regions (arranged in rows), targeted by a 10–20 system transfer method and defined by the “Brodmann” Atlas (PMC-SMA: premotor & supplementary motor cortex, SAC: somatosensory association cortex, M1: primary motor cortex, IPC: inferior parietal cortex; L: left hemisphere, R: right hemisphere; Zimeo Morais, Balardin, & Sato, 2018). D: Experimental procedure: Participants started with a 5 min warm-up phase. Afterwards participants performed BSDT with the dominant right hand (DH), non-dominant left hand (NDH) and dribbling with both hands alternating (AH) in a slow and a fast pace in random order.

The aim of the present study was to compare hemodynamic response alterations during a BSDT with varying levels of complexity including performance with the dominant right hand, the non-dominant left hand and dribbling with both hands alternating. In addition, performance with each hand was performed at slow walking pace (0.87 ms^-1^) and fast walking pace (1.75 ms^-1^), resulting in six conditions: DH_slow_, NDH_slow_, AH_slow_, DH_fast_, NDH_fast_, AH_fast_. In all conditions, walking pace was instructed using a metronome. BSDT was performed in a circular slalom course with a diameter of 10 meters (Fig 1A). In the slow pace conditions (DH_slow_, NDH_slow_, AH_slow_) the participants had to walk to the next slalom bar at every beep (20 bpm). In the fast pace conditions (DH_fast_, NDH_fast_, AH_fast_), the participants had to walk to the next but one slalom bar at every beep (20 bpm) resulting in doubled walking pace. Apart from walking pace, the six conditions differed only with regards to the hand that performed ball dribbling. Each dribbling condition was performed in a block design of 6 x 21 s (with an inter-block rest time of 21 s). The order of conditions was randomized; however, all trials of each condition were performed consecutively in one performance block (Fig 1D). At the end of the experimental procedure, participants rated their perceived level of task complexity of each task on a scale ranging from one to ten. All dribbling movements were additionally recorded with a tripod-supported video camera (Panasonic Lumix DMC-FZ300, Japan, 50 frames/sec). The camera was positioned 5 meter outside of the slalom course, in order to capture the entire course. In order to sufficiently control the comparability of the different conditions, the dribbling frequencies of all participants were counted by means of the video image recordings.

### Functional Near-Infrared Spectroscopy (fNIRS)

Hemodynamic response alterations in sensorimotor areas (M1, PMC, SMA, inferior parietal cortex (IPC), somatosensory association cortex (SAC)) on both hemispheres were assessed using a portable fNIRS system (NIRx Medical Technologies, Glen Head, NY). The fNIRS configuration involved eight light sources and seven detectors with an inter-optode distance of 30 mm, providing a total number of 22 measurement channels (Fig 1B and 1C). Fixation of sources and detectors as well as maintenance of the inter-optode distance was realized by means of distance holders. The applied NIRSport system emits light simultaneously at wavelengths of 760 nm and 850 nm and uses time and frequency multiplexing in order to minimize crosstalk between optodes and wavelengths. Data was recorded with a sampling frequency of 7.81 Hz. Optodes were positioned on a fNIRS cap (in different sizes depending on the participants’ head sizes) according to the established 10–20 system to ensure a standardized optode placement.

With respect to recent recommendations for fNIRS measurements [22, 23], we used an additional short-distance detector bundle (NIRx Medical Technologies, Glen Head, NY) in order to eliminate potential fNIRS confounders such as alterations in extra-cerebral blood flow. The bundle involved additional short-distance detectors for each source with an inter-optode distance of 8 mm, as opposed to the inter-optode distance for all other (long) standard channels of our configuration (30 mm). Hence a total of 8 short-distance channels were considered in the analysis of fNIRS data.

Additionally, the combination of fNIRS neuroimaging and a simultaneous assessment and analysis of systemic physiological signals is very important [24], particularly when hemodynamic responses of different motor tasks are analyzed, comparatively. Hence, we measured heart rate during BSDT with a heart rate monitor watch (RS400, sensor Polar H1, PolarElectro, Kempele, Finland).

### Data analysis

#### Hemodynamics

FNIRS data analysis was performed in MATLAB (MathWorks, Natick, MA, United States of America) using functions provided in the HOMER2 package [22]. Statistical analysis was performed using SPSS Statistics 22 (IBM, Armonk, NY), R 3.4.3 [25] and RStudio 1.1.383 [26]. The fNIRS signal pre-processing steps to reduce the influence of motion artifacts and physiological noise were applied as in Carius et al. [16]. According to this procedure (HOMER2 *PruneChannels* function), only 2.80% of the channels were regarded as too noisy and therefore not included in further analysis steps. Raw intensity signals were converted to changes in optical density [22]. Correction for motion artifacts was performed using a hybrid method that takes advantage of different correction algorithms, so-called Spline interpolation with Savitzky-Golay (SG) filtering [27]. We used the algorithm described by Jahani et al. [27] as implemented in the HOMER2 *hmrMotionCorrectSplineSG* filtering function (*p* = .99, *FrameSize_sec* = 6, [27]). Subsequent to motion-artifact correction, data was band-pass filtered to attenuate low frequency drift, Mayer wave, breathing rate and heart rate components using 0.01 Hz as high and 0.09 Hz as low pass cutoff frequencies [28].

In a further step, attenuation changes of both wavelengths (850 nm and 760 nm) were transformed to concentration changes of oxy- and deoxygenated hemoglobin (ΔHb & ΔHHb, respectively) using the modified Beer-Lambert approach (partial pathlength factor: 6.0; [22]). Although ΔHHb is considered as the more valid parameter to evaluate alterations in hemodynamic response [29], reporting ΔHb and ΔHHb (instead of only one of both) is strongly recommended in current literature and allows better physiological interpretation of the functional experimental results [23].

Extra-cerebral contaminations measured by short-distance channels were regressed out of the fNIRS signal (short separation regression, SSR) using HOMER2 *hrmDeconvHRF_DriftSS* function as previously applied in recent studies [15, 30]. In detail, using this function, SSR is performed with the nearest short separation channel, assuming that the signal measured by short-distance channels represents superficial layers and the signal measured by long-distance channels represents both brain tissue and superficial layers. This approach enables to detect the influence of systemic physiology in superficial layers and to use it as a regressor to filter systemic interferences from long-distance channels in order to provide a more robust estimation of hemodynamic changes underlying brain activation [31].

Finally, single trials of BSDT were baseline corrected (5 seconds until stimulus onset) and time courses of ΔHb and ΔHHb for each channel were block-averaged using HOMER2 *hmrBlockAvg* function. Though the experimental block design included long breaks to prevent overlapping of hemodynamic responses between trials, we directly analyzed the height of amplitude (baseline-corrected average of the temporal window from 5 to 21 seconds with regard to stimulus onset for ball dribbling; [32]).

#### Statistical analyses

Differences regarding task-dependent dribbling frequencies and perceived level of task complexity were analyzed using Friedman tests. Post-hoc Wilcoxon signed-rank test was conducted with Bonferroni correction, resulting in a significance level at p < 0.0083 (.05/6 [number of conditions]).

In order to evaluate contrasts between task-related hemodynamic response alterations during BSDT, dependent t-tests were conducted in a channel-wise manner. We applied *robust statistical tests* [33], since the assumptions for parametric tests are often violated for fNIRS data (e.g., normal distribution). Hence, differences were tested using robust two-sample trimmed mean tests (*yuend* function, trim = 0.2, [33]). To compare hand use differences in terms of ipsilateral and contralateral hemodynamic response alterations, fNIRS data for dribbling with the non-dominant left hand were flipped from the opposite hemisphere across the interhemispheric midline. Differences in ΔHb and ΔHHb between different conditions of BSDT with regards to dominant right, non-dominant left and alternating hand-use, and to slow and fast pace (hand & pace as within-subject-factors) were tested using a robust two-way factorial ANOVA (*t2way* function, trim = 0.2, [33]). We used robust post hoc two-sample trimmed mean tests (*yuend* function, trim = 0.2, [33]) to test specific differences for within-subject factors hand and pace.

These robust statistical tests were conducted in R using the *WRS2* software package [34]. As suggested by Wilcox and Tian [35], an *explanatory measure of effect size ξ* was reported, while Values of ξ = 0.10, 0.30, and 0.50 correspond to small, medium, and large effect sizes. Since *t2way* function does not provide effect sizes, we calculated effect sizes for main effects of hand and pace separately using *t1way* function. Furthermore, false discovery rate (FDR) correction was applied to control for multiple comparisons during robust t-tests [36]. Resulting channel-wise t- und f-values were assigned to the brain surface using Brain Function Mapping Tool by Wang et al. [37]. Using WRS2 package in R, robust correlation coefficients were calculated to correlate perceived level of task complexity with channel-wise ΔHHb during slow (including tasks DH_slow_, NDH_slow_, AH_slow_) and fast (including tasks DH_fast_, NDH_fast_, AH_fast_) basketball slalom dribbling. For all tests, a p-value of < .05 was considered significant.

## Results

### Behavioral data

**Fig 2:**
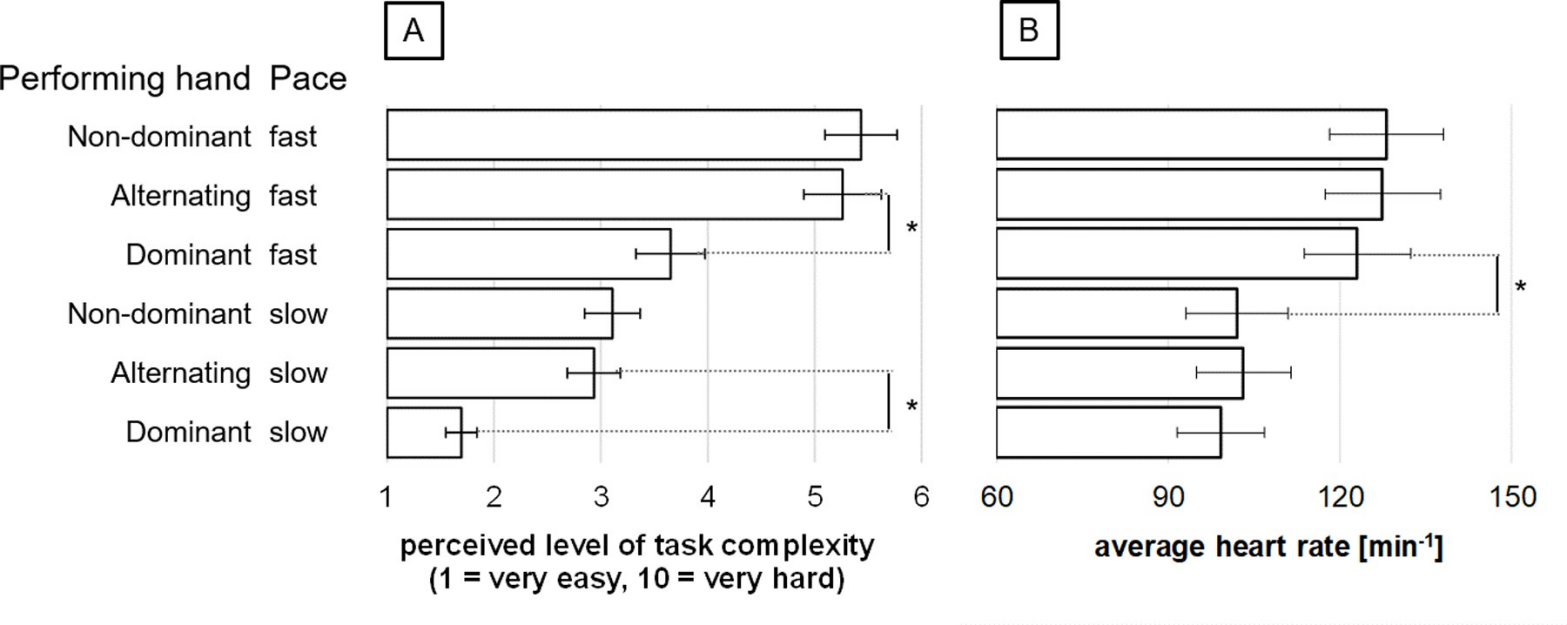
Perceived level of task complexity and cardiac stress during BSDT. A: Perceived level of task complexity (on a scale ranging from 1 = very easy to 10 = very hard) during slow dribbling with the dominant right hand was significantly easier as compared to all other five conditions. In addition, fast dribbling with alternating hands and fast dribbling with the non-dominant left hand were significantly more complex as compared to all other conditions. B: The cardiac stress (heart rate) was significantly higher during fast dribbling conditions as compared to slow dribbling conditions.

Participants performed BSDT with the following dribbling frequencies (Hz): DH_slow_: 1.35 ± .07; NDH_slow_: 1.36 ± .09; AH_slow_: 1.34 ± .09; DH_fast_: 1.35 ± .11; NDH_fast_: 1.37 ± .08; AH_fast_: 1.38 ± .13. There was no difference in dribbling frequencies across conditions (*χ*^*2*^ (5, 21) = 9.55, *p* = .09). The perceived level of task complexity differed for the six BSDT conditions (*χ*^*2*^ (5, 23) = 87.99, *p* < .001). Participants rated DH_slow_ as the task with lowest complexity, followed by AH_slow_, NDH_slow_, DH_fast_, and AH_fast_, NDH_fast_ (Fig 2A).

Average heart rates of all participants during BSDT conditions (DH_slow_: 99.28 ± .49; NDH_slow_: 102.03 ± .58; AH_slow_: 103.15 ± .75; DH_fast_: 123.12 ± .37; NDH_fast_: 128.30 ± .37; AH_fast_: 127.50 ± .33; Fig 2B) changed as a function of movement pace. Cardiac stress/ heart rate was higher during fast dribbling tasks compared to slow dribbling tasks (*F*(2.14, 47.05) = 170.13, *p* < .001, *η*_*p*_^*2*^ = .89). Average heart rate differences showed no interaction of pace (fast vs slow) over the time course of the experiment (*F*_(9.58, 210.71)_ = 1.54, *p* = .13, *η*_*p*_^*2*^ = .06). Additionally, attention (z = -1.06, p = .29) and fatigue (z = -.08, p = .94) did not differ before and after task performance. Regarding pain, there was an increase from 1.22 ±. 52 to 1.61 ± .84 (z = -2.46, p = .01), however, a value of 1.61 can still be considered as a marginal level of pain.

### Hemodynamics

**Fig 3:**
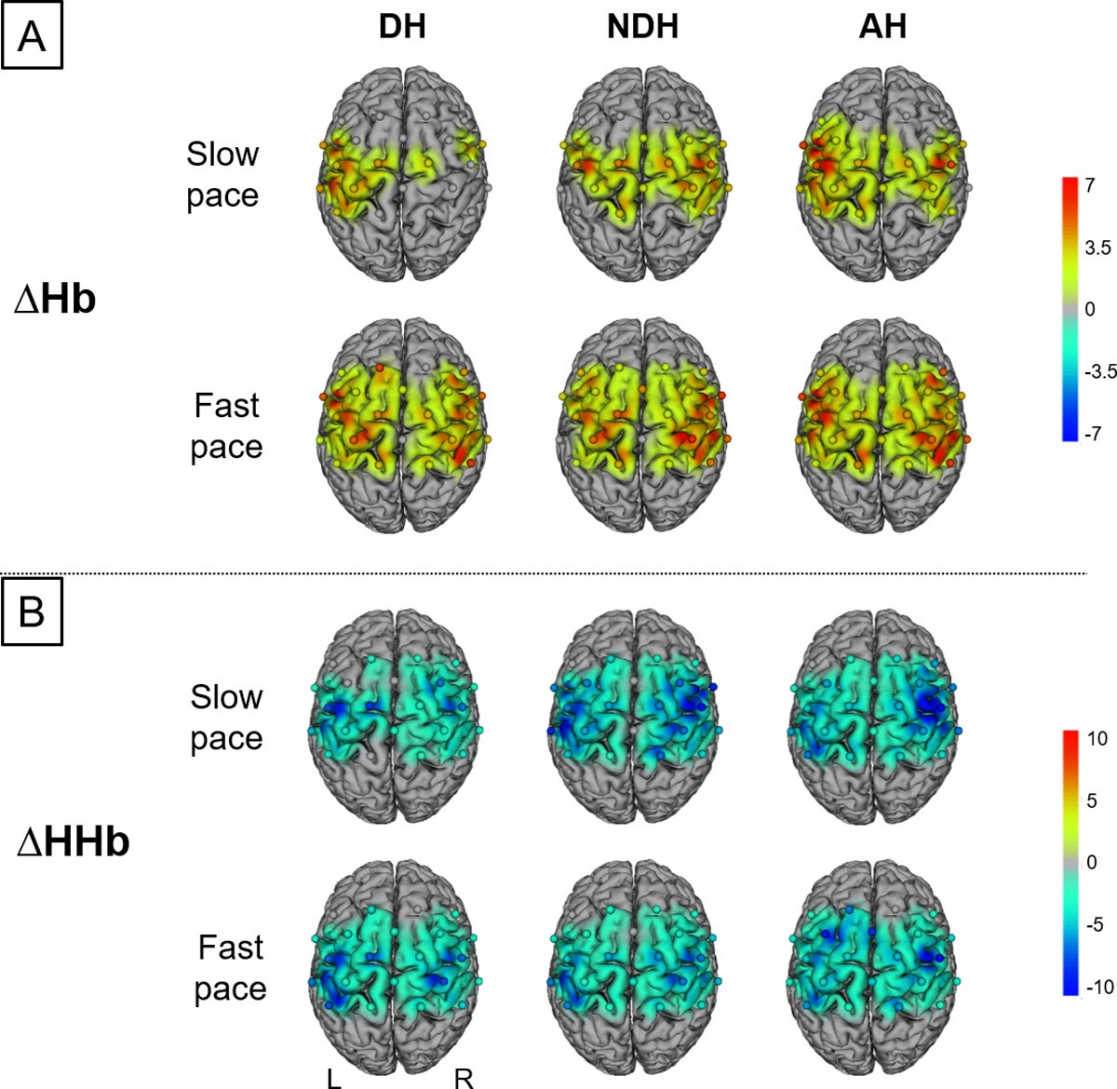
Hemodynamic responses during basketball slalom dribbling task. L: left hemisphere, R: right hemisphere, ΔHb/ΔHHb: oxygenated/ deoxygenated hemoglobin concentration changes. DH: dominant right hand; NDH: non-dominant left hand; AH: alternating hands. A: Illustrates results for changes in ΔHb and B: for changes in ΔHHb; t-maps, robust dependent sample mean tests, Wilcox, 2017; Mair and Wilcox, 2017). Activation maps with cortically projected channel positions (dots centered between source-detector pairs). Only t-values accompanied with FDR corrected p-values are shown.

**Fig 4:**
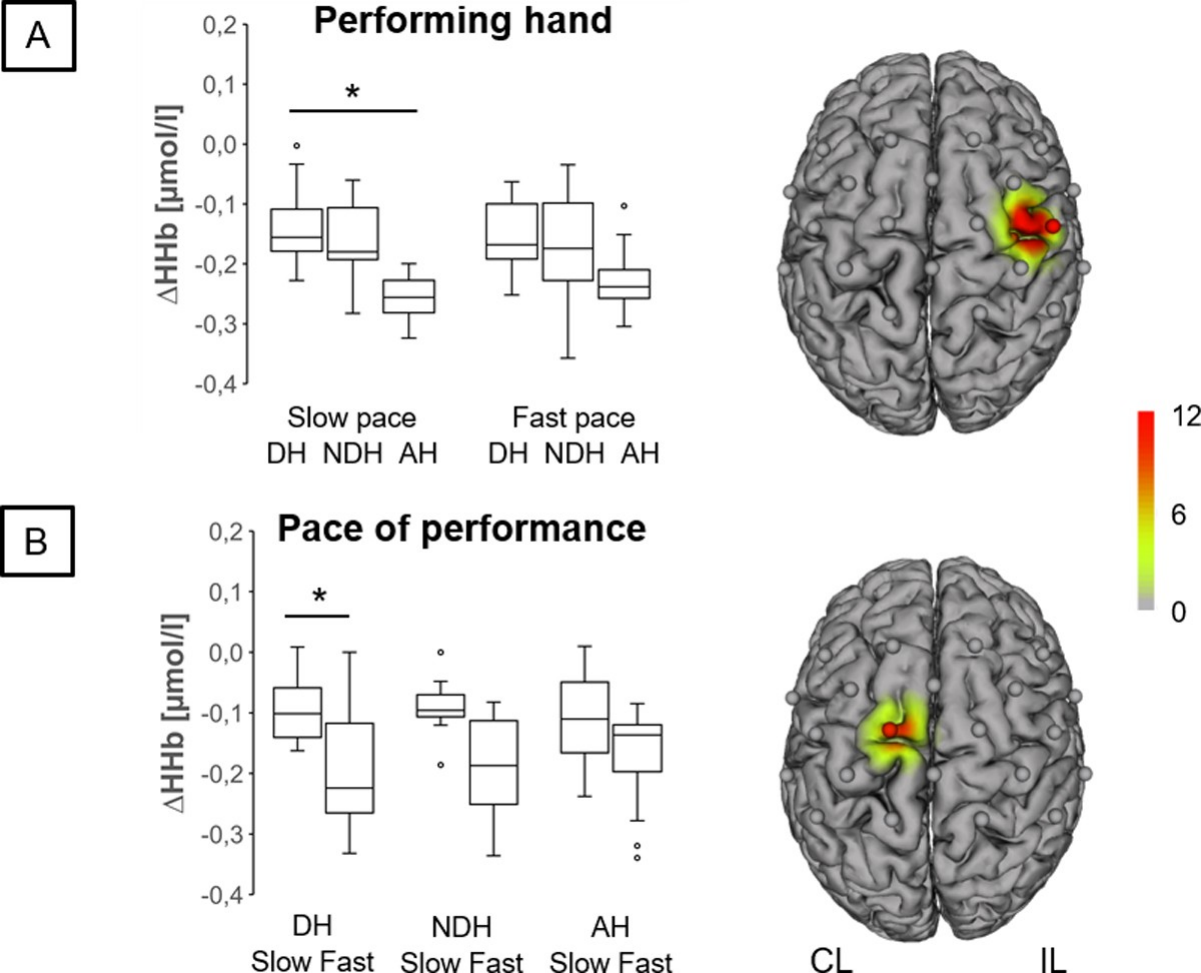
Hand and pace effects on deoxygenated hemoglobin concentration changes (ΔHHb) during basketball slalom dribbling task. DH: dominant hand; NDH: non-dominant hand; AH: alternating hands, CL: contralateral hemisphere, IL: ipsilateral hemisphere, PMC-SMA: premotor and supplementary motor area, M1: primary motor area. Figure illustrates results after short-separation regression. Boxplots represent all participants. Values are median and interquartile range (being the 25th and 75th percentile). Channels (centered between transmitters and detectors) are shown for the topographic image; Note: for dribbling with both alternating hands, the ‘ipsilateral’ hemisphere represents the right hemisphere); colors represent f values. Images are thresholded at p < 0.05 and FDR-corrected (false discovery rate) for multiple comparisons. A: * indicates a significant influence of factor hand on ΔHHb (C2-C4: F(2, 130) = 18.49, p = .001; ξ = .38; p-FDR = .036), indicating a larger ΔHHb decrease across all participants during slow dribbling with alternating hands as compared to slow dribbling with dominant hand in ipsilateral M1 (Post-hoc, C2-C4: t(13) = 3.58, p = .003, ξ = .63). B: * indicates a significant influence of factor pace on ΔHHb (C1-Cz: F(1, 130) = 10.47, p = .002, ξ = .42; p-FDR = .002), indicating a larger ΔHHb decrease across all participants during fast dribbling with dominant hand as compared to slow dribbling with DH in contralateral PMC-SMA (Post-hoc, C1-Cz: t(11) = 3.97, p = .002, ξ = .65).

During BSDT, we found typical hemodynamic response pattern across all conditions (Fig 3). A robust two-way factorial ANOVA revealed significant influences of factor hand in M1 (ΔHHb, C2-C4: *F*_(2, 130)_ = 18.49, *p* = .001; ξ = .38; p-FDR = .036; Fig 4A) and factor pace in PMC-SMA (ΔHb, FC4-FC2: *F*_(1, 130)_ = 16.99, *p* = .001, ξ = .47; ΔHHb, C1-Cz: *F*_(1, 130)_ = 10.47, *p* = .002, ξ = .42; p-FDR = .002; Fig 4B), but no significant hand x pace interactions (ΔHb: 0.13 ≤ *F*_(2,130)_ ≤ 2.49, .302 ≤ p ≤ .938; p-FDR = .002). Post-hoc analyses for factor hand revealed that during DH_slow_ less activation in ipsilateral M1 was found as compared to AH_slow_ (ΔHHb, C2-C4: t(13) = 3.58, p = .003, ξ = .63; Fig 4A). Post-hoc analyses for factor pace revealed less activation during DH_slow_ in contralateral PMC-SMA as compared to DH_slow_ (ΔHHb, C1-Cz: t(13) = 3.97, p = .002, ξ = .65; Fig 4B). Furthermore, less activation was found during AH_slow_ in PMC-SMA as compared to AH_fast_ (ΔHb, FC4-FC2: t(14) = -4.91, p < .001, ξ = .65).

### Correlation between perceived level of task complexity and hemodynamic response alterations

**Fig 5:**
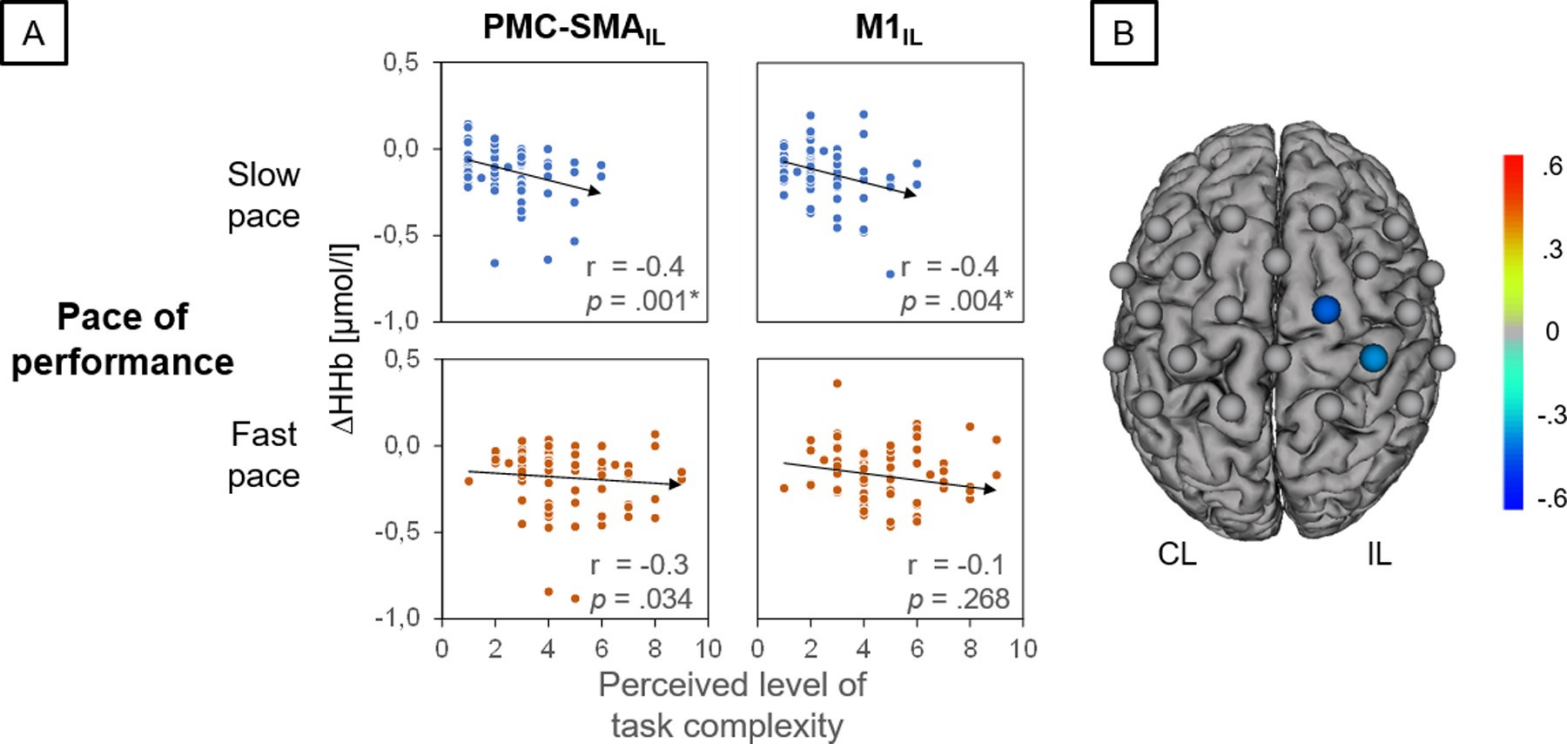
Correlation analysis between perceived level of task complexity and deoxygenated hemoglobin concentration changes. CL: contralateral, IL: ipsilateral hemisphere, PMC-SMA: premotor and supplementary motor area, M1: primary motor area. ΔHHb: deoxygenated hemoglobin concentration changes. A: Figure illustrates results for slow and fast basketball slalom dribbling task. Participants showed a significant negative association within ipsilateral PMC-SMA (C2-Cz) and ipsilateral M1 (C2-CP2) for ΔHHb only during slow basketball slalom dribbling task. Note: A negative correlation for ΔHHb means an increase in concentration changes with increasing perceived level of task complexity. B: fNIRS configuration showing channels with significant negative associations.

Participants showed a significant negative association within ipsilateral PMC-SMA (C2-Cz: r = -.41, p = .001, FDR-corrected, Fig 5A) and ipsilateral M1 (C2-CP2: r = -.35, p = .004, FDR-corrected, Fig 5A) for ΔHHb only during slow dribbling conditions. A negative correlation for ΔHHb indicates an increase in hemodynamic response with increasing perceived level of task complexity. The perceived level of task complexity did not correlate with heart rate during the execution of BSDT (DH_slow_: *ρ*_s_(23) = .10, p = .65; NDH_slow_: *ρ*_s_(23) = .12, p = .60; AH_slow_: *ρ*_s_(23) = .10, p = .64; DH_fast_: *ρ*_s_(23) = .18, p = .40; NDH_fast_: *ρ*_s_(23) = .18, p = .40; AH_fast_: *ρ*_s_(23) = .25, p = .25), indicating that cardiac parameters did not mediate the relationship of perceived level of task complexity and hemodynamic response.

## Discussion

### Hemodynamic responses during basketball slalom dribbling

In the present study, we provided novel evidence for hemodynamic response alterations in sensorimotor brain regions during a complex sport-specific movement. More specifically, we found differential effects of task complexity during basketball dribbling when comparing hand use and pace of execution.

During BSDT, we found activation in bilateral PMC-SMA, M1 as well as IPC and SAC. Moreover, task-specific characteristics with regard to the factor hand use were found for ΔHb during DH_slow_, revealing significant hemodynamic responses for contralateral left hemisphere, in particular. All other conditions (including dribbling with NDH) induced bilateral hemodynamic responses for both ΔHb and ΔHHb. Previous studies explained bilateral M1 activations with the prominent role of right M1 in performing visuo-motor transformation and interhemispheric connections [10]. Furthermore, M1 is known to be essential for planning, preparation and execution of complex movements [38], which explains its hand use independent activation. Moreover, activations of left dorsal premotor areas especially during task execution with NDH might be relate to the fact that premotor regions are used when planning and execution of complex motor sequences are necessary, independent of the executing hand [2]. Furthermore, according to Halsband, Ito, Tanji, and Freund [39], especially left-hemispheric PMC and SMA are relevant regions regarding the temporal aspects of motor programming, particularly in alternating movements. Hence, it is reasonable to assume that motor and somatosensory brain regions are crucial for the execution of basketball dribbling, although we cannot exclude the involvement of other brain regions outside sensorimotor areas such as the cerebellum, subcortical and/ or prefrontal networks.

### Influence of pace and laterality on basketball slalom dribbling

We also hypothesized, that the level of hemodynamic response is related to movement pace in BSDT, such that lower brain activation is observed during slow dribbling as compared to fast dribbling condition. In fact, we found significantly lower brain activation during DH_slow_ in contralateral PMC-SMA regions as compared to DH_fast_. Although the number of investigations focusing on the influence of walking pace on the fNIRS signal is limited, previous studies revealed similar findings [7, 40]. For example, it has been shown that an increase in walking pace is related to an increased PFC [40] and SMA [7] activation. While Metzger et al. [8] revealed an increased brain activation during faster walking conditions on both hemispheres, findings of Harada et al. [7] indicate that particularly left prefrontal cortex (PFC), SMA and SMC might be responsible for the control of walking pace, going in line with our findings of particularly left PMC-SMA activation. Study findings regarding the neural activation patterns of walking pace were recently summarized in a review by Vitorio, Stuart, Rochester, Alcock, and Pantall [41]. This review mainly concluded, that observed increases in brain activation in relation to walking pace might originate mainly from methodological issues that favor the influence of movement artifacts [41]. In this regard, however, our study is designed according to recent recommendations on how to reduce movement artifacts [27, 42, 43].

We further hypothesized, that dribbling with DH would lead to lower ipsilateral brain activation as compared to dribbling with NDH, which was not confirmed by our results. This finding is contrarily to a previous study by Alahmadi et al. [10], comparing brain activations during a power grip task executed with DH and NDH. According to the authors, execution with NDH is associated with an increased activation not only in areas involved in movement control, but also in cognitive regions such as hippocampus and PFC, which are known to be relevant for increased attentional demands. Thus, we assume, that differences between basketball slalom dribbling with DH and NDH are rather present in brain regions not covered by the fNIRS configuration used in the present study. However, although previously expected for both hemispheres, we found less brain activation in ipsilateral M1 during DH_slow_ dribbling as compared to AH_slow_ dribbling. According to Alahmadi et al. [10], this might be due to a crucial function for the ipsilateral M1 in performing unimanual DH tasks in right-handed participants. Authors assume, that right M1 processes visual feedback signals and translates it into movement, which is consistent with the dominant role of the right hemisphere in visually guided movements. Due to the exploratory character of the present study, we can furthermore only speculate about a kind of a ceiling effect for task-related differential cortical changes. This speculative assumption is based on the fact, that even though tasks were rated as being more demanding, no differential cortical changes were observed. However, future studies need to investigate to what extent this assumption is true and whether this observation can also be made in other task-relevant brain regions.

### Perceived level of task complexity is associated with hemodynamic response alterations

Additionally, a correlation analysis investigating the relationship between hemodynamic response alterations and perceived level of task complexity was performed. We found an increase in ΔHHb (i. e. an increase in brain activation) to be associated with an increased perceived level of task complexity during slow conditions. This finding can be explained by assuming that participants with a lower perceived level of task complexity are those with better BSDT skills. Based on this assumption, our result suggests that participants with better skills in basketball slalom dribbling require less neural resources during task execution as compared to less skilled participants, which in turn supports the “neural efficiency” hypothesis as proposed by Dunst et al. [44]. While this hypothesis was originally investigated in intelligence research, similar results were also found in recent studies focusing on athletes such as table tennis players [45] and endurance athletes [46], showing less brain activation during task execution as compared to non-athletes/ novices. However, since individual skill levels of participants in relation to the task were only indirectly assessed, the interpretation of this finding needs to be considered with caution. Approaches for future studies in order to verify the “neural efficiency” hypothesis also in the context of basketball are discussed in the following limitation section.

### Study limitations

In the present study, we used a multi-distance fNIRS approach to observe task-related hemodynamic response alterations during the execution of a BSDT. However, fNIRS only allows the observation of hemodynamics in superficial cortical brain regions. Therefore, fNIRS findings must always be interpreted with caution, since penetration depth of near-infrared light is only ~ 1.5 cm [47, 48]. Hence, subcortical structures cannot be investigated while performing complex sport-specific motor tasks using this imaging technique. Furthermore, task-related effects regarding differential hemodynamic response alterations were only assessed in central motor areas. However, recent studies have shown that different task complexity is particularly associated with prefrontal alterations [49]. Therefore, for future studies, it is reasonable to suggest the use of whole-brain fNIRS configurations in order to get a full holistic view of task-related hemodynamic response alterations. Additionally, the investigation of expertise effects provides an interesting issue for future research, which was not addressed in the present study, since only novices were included. Future studies should aim at objectively operationalizing motor expertise in basketball slalom dribbling in order to sufficiently evaluate performance of participants. Potential differences between experts and novices as well as particularities of experts would probably allow further sport-specific conclusions.

## Conclusions

Taken together, this study provides novel evidence that multi-distance fNIRS is a valid and valuable tool to assess hemodynamic response alterations during the execution of complex, non-stationary motion sequences. We revealed that sensorimotor brain regions are involved in basketball slalom dribbling and that these regions show functional plasticity as a function of hand use and pace of movement. Furthermore, we showed that lower levels of perceived task complexities are associated with less neural activation in motor related brain regions, which indirectly indicates that participants with better BSDT skills require less neural resources as compared to less skilled participants. These findings provide a first step towards understanding, how performing a complex motor task such as playing basketball is represented in the human motor system. This knowledge is of particular interest in many sports disciplines, since quantifying brain processing might be crucial for efficient motor control and sport-specific skills. Further implications are not limited to sports, but are also conceivable in the field of rehabilitation, e. g. after surgery, stroke or neuromuscular diseases. Hence, in the future, knowledge on performance-relevant brain regions and task-related activation pattern should be extended to further complex movements and sports disciplines in order to obtain a profound understanding of the brain-behavior relationship.

## Supporting information

S1 Data (SAV)
